# Women’s health and primary care: time to get it right for the life course

**DOI:** 10.3399/bjgp21X717713

**Published:** 2021-11-26

**Authors:** Sharon Dixon, Abigail McNiven, Anne Connolly, Lisa Hinton

**Affiliations:** Nuffield Department of Primary Care Health Sciences, University of Oxford, Oxford.; Nuffield Department of Primary Care Health Sciences, University of Oxford, Oxford.; Bevan House Healthcare, Bradford; Chair, Primary Care Women’s Health Forum; Clinical Champion, Royal College of General Practitioners Women’s Health.; The Healthcare Improvement Studies Institute, University of Cambridge, Cambridge.

Recent Women’s Health Strategies, published in Scotland^1^ and announced for England,^2^ are a welcome recognition that for too long women have lived within health and care systems designed mostly for men, by men.^2^ Their explicit focus on women’s health needs throughout the life course is sorely needed.

Women face significant health inequalities across their lives. Although women typically live longer than men, fewer of those years are in good health.^2^ Those living in social and economically deprived settings experience even poorer outcomes.^3^ These inequalities have been exacerbated by the COVID-19 pandemic, including increased period poverty,^4^ increased domestic violence,^5^ and women carrying a greater burden of home schooling and unpaid care work.^6^

## WHAT COULD (OR SHOULD) THIS MEAN FOR PRIMARY CARE?

Primary care needs to be at the heart of any strategy to support and enhance women’s health. Primary care can (and does) play a central role in supporting women from before menarche to the menopause and beyond. GPs support women who might never need (or want) specialist input from secondary care. However, even where women are supported in secondary care, the GP’s role encompasses care before, during, and beyond periods of specialist or focused secondary care involvement.

The GP role includes prevention as well as early intervention including through contraception, pre-conception advice and screening, and menopause management.

## PROBLEMS WITH RESEARCH IN WOMEN’S HEALTH

Historically, women have been underrepresented in trials and research data.^7^ This has led to a medical understanding of what is usual in health and symptomatology, or in health service development and design, that is male by default and not based on a knowledge of women’s differences and needs.^8^ A striking illustration of the potential harms of these inequalities is women’s experience in ischaemic heart disease where they are said to experience atypical symptoms of heart disease (that is the symptoms differ to those that men typically experience and describe, which form the basis of medical education and training). The failure to recognise the prevalence of heart disease in women and the different set of symptoms they experience during a heart attack contribute to delays in help seeking and the loss of valuable time in a cardiovascular emergency.^9^

Alongside the relative lack of representation of women in research of conditions affecting both men and women, conditions that pre-dominantly affect women have been historically under-researched.^10^ Endometriosis is an important example of this, as a long-term debilitating chronic condition affecting 6%–10% of women, which is significantly under-researched compared with other long-term conditions with comparable incidence (such as diabetes).^11^

## LACK OF RESEARCH ON WOMEN’S HEALTH IN PRIMARY CARE

Evidence and resources that understand the role of primary care in women’s health and recognise the complexity of what primary care can and does do, are needed in order to optimise this capability and potential. Women’s health is often narrowly focused on reproductive health but, in primary care, GPs care for all of women’s health needs throughout the whole life course. Evidence and resources that can inform and support holistic and longitudinal care are needed yet currently lacking in many areas of women’s health.

In our work on endometriosis^12^ and female genital mutilation (FGM),^13^^–^^14^ we found that the majority of evidence deployed in primary care is derived from specialist settings and then extrapolated back to the primary care setting, where the populations and needs may significantly differ.

Most women across the spectrum of women’s health (before adolescence to menopause and beyond) are cared for exclusively in primary care, with a relatively small number of women referred for specialist care. Those who are referred are more likely to have symptoms that are difficult to understand or manage, or more complex health needs. Using evidence and guidance predominantly derived from specialist settings presents a denominator problem for primary care clinicians trying to determine risk, share decisions, or advise on management options, where the knowledge and evidence relates to a different population from the one they are working with. For example, the National Institute for Health and Care Excellence guidance on endometriosis suggests that referral for specialist care is considered if symptoms are not controlled with first-line therapies such as hormonal treatment or non-steroidal anti-inflammatory drugs.^15^ This leaves GPs facing uncertainty about how to support women with impactful period pain, whose symptoms are well controlled with first-line therapies, against the backdrop of widespread reporting of delayed referrals to specialist endometriosis clinics.^12^

Another example is the predominance of FGM research in obstetric and midwifery settings resulting in a lack of evidence or resources for how GPs might support women with FGM beyond their reproductive years and through the menopause.^14^ Where potential gaps in care are identified, all too often the conclusion is that GPs lack knowledge and awareness, and that increasing these would improve care.^12^ However, our work on endometriosis demonstrates GPs are rarely working with a lack of knowledge, but rather engage with complex and nuanced considerations. They are already balancing multiple possibilities and involved in complex shared decision making with women based on knowledge about known uncertainties and the challenges at the primary to secondary care interfaces.

## CONCLUSION

These new Women’s Health Strategies offer opportunities to put primary care at the heart of enhancing women’s health throughout the life course. But to achieve this we need evidence and knowledge developed with, from, and for primary care.

We need to ensure that the services and resources developed in response to these strategies do not become too symptom or condition specific, risking compartmentalising women’s lives and bodies into organs, conditions, and phases of life. Instead, this is an opportunity to call for primary care focused resources, education, and services that will enable GPs to support patients throughout the life course, and across their physical, psychological, and social wellbeing needs.

Within primary care, there are opportunities to identify and mitigate against health inequalities in women’s health, which would benefit all of society.

Primary care’s huge strength is being there for the journey. It would be a missed opportunity if the conclusion and outcome of these consultations defaulted to an explanation of ignorance and to pillorying GPs to simply know more.

Instead, we urge policymakers to positively utilise the wisdom and experience of GPs and patients, in research and consultation, to support an effective and meaningful women’s health strategy inclusive of primary care.
